# Lipid metabolism reprogramming shapes the immune landscape in the tumor microenvironment

**DOI:** 10.1038/s41423-026-01411-0

**Published:** 2026-04-07

**Authors:** Yun-Wei Du, Ze-Rong Cai, Xiao-Tong Duan, Xin-Yu Li, Tong Yue, Tian Tian, Jian-Jun Li, Huai-Qiang Ju

**Affiliations:** 1 State Key Laboratory of Oncology in South China, Guangdong Provincial Clinical Research Center for Cancer, Sun Yat-sen University Cancer Center, Guangzhou, PR China; 2 Department of Medical Biochemistry and Molecular Biology, School of Medicine, Jinan University, Guangzhou, PR China

**Keywords:** Lipid metabolism reprogramming, antitumor immunity, immune evasion, tumor microenvironment, Cancer metabolism, Cancer microenvironment, Immunosurveillance

## Abstract

Given the fundamental biological importance of lipids not only as structural components and energy substrates but also as potent bioactive molecules that govern immune and oncogenic signaling, lipid metabolism reprogramming has emerged as a central driver of tumor progression. Rather than merely fueling tumor growth, this extensive metabolic rewiring profoundly reshapes the tumor microenvironment (TME), establishing complex metabolic crosstalk that actively drives immune evasion. This review examines the current understanding of lipid metabolism reprogramming across different cellular compartments within the TME and its far-reaching implications for cancer immunotherapy. We first delineate how altered lipid metabolism directly fuels tumor cell proliferation, survival, and metastatic potential. We then examine the distinct lipid metabolic patterns in different immune cells, detailing how this reprogramming drives dysfunction in antitumor subsets such as CD8^+^ T cells and natural killer cells and how it promotes immunosuppressive populations such as tumor-associated macrophages and myeloid-derived suppressor cells. In addition to these immune alterations, we address the metabolic rewiring of stromal cells, particularly cancer-associated fibroblasts. Furthermore, by exploring intricate intercellular crosstalk, we highlight how tumor lipid metabolism promotes immune escape and how lipids from reprogrammed immune and stromal cells, in turn, support tumor growth, thereby reinforcing an immunosuppressive niche. Finally, we highlight emerging therapeutic strategies targeting these pathways and discuss how leveraging multiomics advances can translate lipid insights into cancer immunotherapy.

## Introduction

Cancer imposes a substantial global health burden, with high incidence and mortality rates. Although chemotherapy and targeted therapies remain the main treatments for advanced-stage disease, their clinical efficacy is often limited [1, 2]. The advent of immunotherapy has led to the introduction of novel approaches that focus on modulating interactions between tumor cells and immune cells. However, immune checkpoint monotherapy, mainly blockade of the programmed death ligand 1 (PD-L1) and programmed death 1 (PD-1) pathways, has yielded modest response rates [3], highlighting the need for more effective strategies. Recently, a phase II trial involving patients with treatment-refractory, microsatellite-stable/proficient mismatch repair (MSS/pMMR) colorectal cancer (CRC) demonstrated that a triple regimen comprising chidamide, sintilimab, and bevacizumab significantly improved 18-week progression-free survival [4]. These findings underscore the value of targeting different steps in the cancer-immunity cycle. However, an insufficient mechanistic understanding of immune evasion in tumors, particularly the role of metabolic reprogramming within the tumor microenvironment (TME), restricts the rational design of combination therapies.

Metabolic reprogramming is a hallmark of cancer, allowing tumor cells to adapt to nutrient-deprived and hypoxic microenvironments while sustaining bioenergetic and biosynthetic demands associated with uncontrolled proliferation. Malignant transformation is accompanied by a broad metabolic shift encompassing not only glucose, lipid and amino acid [5] but also several auxiliary pathways, such as nicotinamide adenine dinucleotide phosphate (NADPH) [6] and iron [7] metabolism. Key nodes of lipid metabolism, including uptake, de novo synthesis, storage, and oxidation, are extensively reprogrammed across multiple cell compartments. For instance, the activation of sterol regulatory element-binding proteins (SREBPs) drives the upregulation of key metabolic enzymes (e.g., ACLY, FASN, and HMGCR) [8–10], enabling tumor cells to coopt de novo lipogenesis and cholesterol synthesis, which secures the supply of structural building blocks required to sustain tumor expansion. Concomitantly, they remodel the phospholipid composition to modulate membrane signaling [11, 12] and adjust triacylglycerol turnover [13–15] to balance bioenergetic and redox homeostasis. This metabolic rewiring extends beyond tumor cells to orchestrate the entire TME, for example, by skewing immune cells toward dysfunctional or immunosuppressive phenotypes to promote immune evasion.

Given the eight lipid categories defined by the LIPID MAPS consortium [16, 17], the immense complexity of the lipidome precludes comprehensive coverage. Therefore, to provide an in-depth discussion, this review focuses on the core metabolic axes of several highly abundant lipid groups centrally influencing the TME. We specifically emphasize fatty acids, cholesterol, triacylglycerols, and membrane phospholipids (such as phosphatidylcholine and phosphatidylethanolamine), given their well-established roles in modulating tumor progression and antitumor immunity. Other lipid species, such as isoprenoids and ceramides, have important context-dependent functions and are briefly mentioned where relevant, although their systematic discussion lies beyond the scope of this review. In the following sections, we first delineate how lipid metabolism reprogramming promotes malignant behaviors and examine its distinct manifestations in immune cells and stromal cells. We subsequently discuss how lipid metabolism–mediated intercellular crosstalk reinforces an immunosuppressive niche. Finally, we highlight emerging pharmacological agents targeting lipid metabolism and multiomics technologies aimed at characterizing this metabolically driven immune landscape.

## Lipid metabolism reprogramming in the tumor microenvironment

**Fig. 1:**
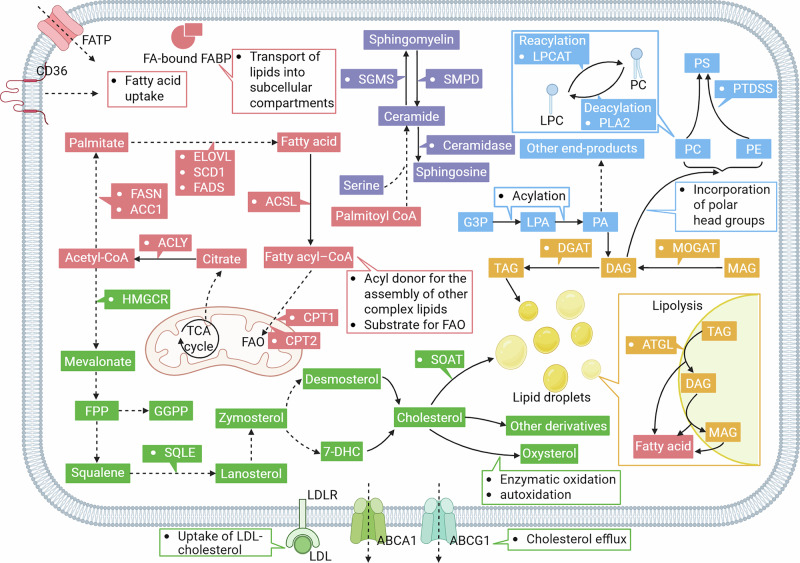
General overview of the core intracellular lipid metabolic pathways. Extracellular lipid resources, such as fatty acids (FAs) and low-density lipoprotein (LDL)–cholesterol, are internalized via transporters and receptors, including CD36 and the LDL receptor (LDLR). These exogenous inputs converge with endogenously synthesized lipids from de novo lipogenesis and the mevalonate pathway, processes orchestrated by key enzymes, such as ATP-citrate lyase (ACLY), fatty acid synthase (FASN), and 3-hydroxy-3-methylglutaryl-CoA reductase (HMGCR). Fatty acyl–CoAs represent central metabolic intermediates, channeled toward energy production via fatty acid oxidation (FAO) or the assembly of complex lipids. Triacylglycerol (TAG) metabolism involves dynamic synthesis and lipolysis within specialized lipid droplets. In parallel, glycerophospholipids, such as phosphatidylcholine (PC) are synthesized by the addition of polar head groups to lipid backbones and undergo remodeling via deacylation–reacylation cycles. Additionally, palmitoyl-CoA and serine serve as the foundational substrates for the de novo synthesis of sphingomyelin (SM). Created at https://BioRender.com. Abbreviations: 7-DHC 7-dehydrocholesterol, ACC acetyl-CoA carboxylase, ACLY ATP citrate lyase, ACSL acyl-CoA synthetase long-chain, ATGL adipose triglyceride lipase, CPT carnitine palmitoyltransferase, DAG diacylglycerol, DGAT diacylglycerol O-acyltransferase, ELOVL fatty acid elongase, FADS fatty acid desaturase, FASN fatty acid synthase, FPP farnesyl pyrophosphate, G3P glycerol-3-phosphate, GGPP geranylgeranyl pyrophosphate, HMGCR 3-hydroxy-3-methylglutaryl-CoA reductase, LDL low-density lipoprotein, LDLR low-density lipoprotein receptor, LPA lysophosphatidic acid, LPC lysophosphatidylcholine, LPCAT lysophosphatidylcholine acyltransferase, MAG monoacylglycerol, MOGAT monoacylglycerol O-acyltransferase, PA phosphatidic acid, PC phosphatidylcholine, PE phosphatidylethanolamine, PLA phospholipase A, PS phosphatidylserine, PTDSS phosphatidylserine synthase, SGMS sphingomyelin synthase, SMPD sphingomyelin phosphodiesterase, SQLE squalene epoxidase, TAG triacylglycerol, TCA tricarboxylic acid cycle

Lipid metabolism reprogramming enables tumor cells to thrive within the challenging TME. This metabolic rewiring is dynamically driven by the integrated effects of intrinsic oncogenic signals and extrinsic microenvironmental factors. Oncogenic alterations [10, 18–21] reshape lipid metabolic pathways at the transcriptional and posttranscriptional levels, while microenvironmental cues, such as growth factors, nutrient availability, and metabolic stresses, such as hypoxia further sculpt this metabolic network. This reprogramming manifests across various facets of lipid handling within tumor cells and further extends to orchestrate the functions of immune and stromal populations within the TME. The fundamental fluxes of intracellular lipid metabolism are shown in Fig. 1, highlighting key enzymes discussed throughout this review.

### Lipid metabolism reprogramming in tumor cells

Reprogramming of fatty acid uptake, lipogenesis, and oxidation critically drives tumor progression in the TME. Excess fatty acids derived from heightened uptake and synthesis are typically sequestered into lipid droplets as energy reservoirs. Beyond this role, lipid droplet accumulation facilitates the recruitment of the E3 ubiquitin ligase MDM2, thus promoting the degradation of the tumor suppressors p53 [22] and Numb [9]. Compositionally, lipid unsaturation levels modulate intracellular metabolic stress to sustain tumor cell survival [23–26]. As exemplified during matrix detachment, AMP-activated protein kinase (AMPK)-mediated CD36 induction drives the selective uptake of monounsaturated fatty acids (MUFAs) to alleviate endoplasmic reticulum (ER) stress, thereby ensuring tumor cell survival during metastatic dissemination [24]. Recent studies link increased invasiveness to a metabolic shift from stearoyl-CoA desaturase 1 (SCD1) to the fatty acid desaturase FADS2 [27, 28], in which FADS2-derived n-10 MUFAs drive epithelial–mesenchymal transition (EMT). Similarly, activated mitochondrial FAO contributes multifunctionally, chiefly by generating ATP, maintaining redox homeostasis, and regulating oncogenic signaling via acetyl-CoA flux [5, 29], thereby promoting tumor proliferation, survival and EMT. Notably, oncogenic alterations can override AMPK-mediated antagonism between lipogenesis and FAO. In IDH1-mutant cancer cells, for example, elevated FAO is maintained independently of AMPK activation but in an ACC1-dependent manner [19].

Remodeling of triacylglycerol synthesis and lipolysis enhances cancer hallmarks chiefly by coordinating intracellular signaling and maintaining energy homeostasis. The upregulation of diacylglycerol O-acyltransferases (DGATs) drives triacylglycerol synthesis, compartmentalizing potentially toxic lipids, such as polyunsaturated fatty acids (PUFAs) into lipid droplets. This sequestration not only maintains lipid homeostasis and restricts lipid peroxidation [13, 14] but also sustains PI3K/AKT/MYC signaling [30], thereby bolstering tumor cell stemness and proliferation while conferring resistance to ferroptosis and apoptosis. The triacylglycerol precursor diacylglycerol functions as a second messenger to stimulate proliferative signaling pathways, including the PKC/MEK/ERK cascade, an axis amplified by hypoxia-inducible factor 1-alpha (HIF-1α) driving diacylglycerol synthesis from monoacylglycerol [31]. Adaptation to metabolic stress within the TME depends on dynamic lipolysis [15, 32, 33]. Under nutrient deprivation, the activation of lipolysis yields fatty acids that fuel FAO [32] and sustain proliferative mTORC1 signaling [33]. In contrast, hypoxia-induced lipid droplet-associated protein HILPDA inhibits adipose triglyceride lipase (ATGL) to restrict fatty acid flux [15], which prevents toxic ceramide accumulation and lipid peroxidation, thereby shielding tumor cells from apoptosis and sustaining survival.

Rewiring of phospholipid metabolism promotes tumor progression by modulating membrane components and generating bioactive signaling lipids. This modulation occurs, fundamentally, through de novo synthesis to support membrane biogenesis during rapid proliferation [34]. Simultaneously, enzymes, such as long-chain acyl-CoA synthetases (ACSLs) [11, 12, 35, 36] and lysophosphatidylcholine acyltransferases (LPCATs) [37, 38] remodel existing phospholipids (especially phosphatidylcholine and phosphatidylethanolamine), chiefly altering membrane properties. For instance, increased incorporation of PUFAs into phospholipids increases membrane fluidity and fine-tunes lipid raft function, thus promoting invasion and migration [11, 12]. Conversely, under metabolic stress, such as ferroptosis, the restriction of lipid peroxidation by reducing the levels of PUFA-containing phospholipids enables tumor survival [35–38]. Maintaining sphingomyelin homeostasis represents another layer of survival adaptation in tumor cells [39–41]. For example, the activity of the lysosomal acid sphingomyelinase SMPD1 preserves lysosomal stability and lipid raft architecture, thereby preventing autophagy-dependent cell death and sustaining pro-survival signaling [39, 40]. In addition to its structural role, rewired phospholipid metabolism generates diverse bioactive molecules involved in oncogenic signal transduction, such as lysophosphatidylserine [42] and phosphatidic acid [43]. More recently, C26-ceramide, a sphingomyelin precursor generated by the ceramide synthase CERS3, was shown to activate the epidermal growth factor receptor (EGFR) in CRC, thereby sustaining proliferative signaling [44].

Emerging evidence highlights the pivotal role of cholesterol accumulation in cancer progression. In general, cholesterol has location-specific functions, such as supporting oncogenic signaling in membrane lipid rafts [45, 46], supporting the activation of mTORC1 in the lysosomal membrane [47], and regulating cell death in mitochondria [48]. Moreover, byproducts of cholesterol synthesis, such as mevalonate-derived geranylgeranyl pyrophosphate (GGPP) [18] and 7-dehydrocholesterol (7-DHC)–derived calcitriol [49], potentiate MAPK signaling through distinct mechanisms, thereby supporting proliferation. In ARID1A-mutant tumor cells, GGPP promotes RhoA prenylation to suppress caspase-1 activity and prevent pyroptosis [21]. Tumor cells also employ context-dependent cholesterol acquisition strategies to support organ-specific metastasis. Upregulation of low-density lipoprotein receptor (LDLR) expression promotes liver colonization in CRC and pancreatic cancer [50, 51]. Mechanistically, cholesterol uptake triggers the degradation of squalene epoxidase (SQLE), thus disrupting the GSK3β/p53 complex and activating β-catenin signaling while suppressing p53 [50]. Conversely, reliance on cholesterol synthesis results in an increase in the levels of cholesterol precursors, such as 7-DHC [51]. This antioxidant lipid suppresses ferroptosis, thus promoting pulmonary metastasis [51, 52]. Thus, lipid metabolism reprogramming in tumor cells promotes their survival, proliferation and metastatic potential by regulating membrane properties, energy metabolism, redox balance and oncogenic signaling.

### Lipid metabolism reprogramming in immune cells

Within the TME, lipid metabolism reprogramming profoundly shapes immune cell functions, ultimately tilting the immune balance toward suppression through the differential regulation of protumor and antitumor populations. In general, immunosuppressive populations, such as M2-like tumor-associated macrophages (TAMs) [53] and regulatory T cells (Tregs) [54], actively accumulate lipids to support their functions. In contrast, in CD8⁺ T cells and natural killer (NK) cells, prolonged immune and metabolic signals cause lipid metabolism dysregulation, which impairs their antitumor potency [55]. Chronic antigen signaling is the main contributor to CD8⁺ T-cell exhaustion, a dysfunctional state characterized by sustained expression of coinhibitory receptors, diminished proliferative capacity, and loss of effector functions [56, 57]. Recent studies have revealed the role of lipid metabolic programming in promoting CD8^+^ T-cell exhaustion.

#### CD8^+^ T cells

Dysregulated fatty acid metabolism in tumor-infiltrating CD8^+^ T cells fundamentally impairs their antitumor capacity. While efficient mitochondrial FAO is essential for sustaining T-cell longevity and polyfunctionality [58–62], this program is dampened by hostile extrinsic stressors within the TME. For instance, the synergistic impact of persistent antigen stimulation, hypoxia, and glucose deprivation promotes the perpetual activity of acetyl-CoA carboxylase 1 (ACC1) to block FAO, leading to energy crisis and accelerated T-cell exhaustion [59]. Similarly, under glucose scarcity, disruption of the surface trafficking of the lipid chaperone FABP5 induced by ER stress impairs lipid uptake and mitochondrial fitness, which compromises memory formation and cytolytic activity while accelerating T-cell exhaustion [61]. Nevertheless, the role of FAO is context dependent, as chronic overactivation can paradoxically impair T-cell function. Driven by leptin and PD-1 signaling, CD8^+^ T cells shift toward FAO at the expense of glycolysis, decreasing the expression of cytolytic effectors, such as IFNγ and granzyme B [63]. This forced metabolic deviation can be accompanied by CD36 upregulation [64], which precipitates ferroptosis via PUFA uptake [65]. Beyond extrinsic metabolic constraints, intrinsic lipid synthesis upon priming causes maladaptation in the TME [66, 67], adding another layer to T-cell dysfunction. Specifically, heightened activity of the fatty acid elongase ELOVL1 represses T-cell receptor signaling and mitochondrial fitness by constraining cholesterol synthesis [66]. Simultaneously, a surge in oleic acid synthesis triggers lipocalin-2 secretion, recruiting myeloid-derived suppressor cells (MDSCs) to reinforce an immunosuppressive milieu [67].

Aberrant phospholipid and cholesterol metabolism further drives CD8^+^ T-cell dysfunction through diverse mechanisms. Disturbances, such as phospholipid synthesis deficiency [68] and cholesterol overload [69, 70] induce secondary stresses, such as oxidative stress and ER stress, which are among the mechanisms that promote canonical CD8^+^ T-cell exhaustion. In addition, dysregulated lipid metabolism impairs CD8^+^ T-cell function through distinct pathways. For example, in a phosphoethanolamine-enriched TME, its enhanced conversion into phosphatidylethanolamine within CD8^+^ T cells depletes the second messenger diacylglycerol, thereby impeding immune activation [71]. Dysregulated cholesterol metabolism is particularly detrimental [72–74]. For instance, excessive cholesterol synthesis triggered by the DNA damage response promotes a senescent state in CD8^+^ T cells [72]. Conversely, cholesterol depletion caused by environmental oxysterols that dysregulate SREBP2 and liver X receptor (LXR) pathways disrupts TCR clustering, attenuates downstream mTOR signaling and induces autophagy-mediated apoptosis [73]. In terms of intrinsic oxysterol biosynthesis within CD8^+^ T cells, tumor-derived prostaglandin E2 (PGE2) downregulates cholesterol-25-hydroxylase (CH25H) via the transcription factor ATF3, resulting in trogocytosis-related apoptosis by reducing essential 25-hydroxycholesterol [75]. Collectively, these findings indicate that lipid metabolism dysregulation converges on critical vulnerabilities, including TCR signaling, energy metabolism, and cell death, thereby driving severe dysfunction in tumor-infiltrating CD8^+^ T cells.

#### NK cells

The TME severely restricts NK cell activity, particularly through imposing metabolic constraints that lead to a loss of “metabolic flexibility” [76]. This manifests as a shift toward a lipid storage phenotype, driven by a detrimental circuit involving CD36, fatty acid binding proteins (FABPs) and peroxisome proliferator-activated receptors (PPARs) [77, 78]. Hyperactivation of this pathway suppresses mTORC1 signaling, downregulates both glycolysis and mitochondrial oxidative phosphorylation (OXPHOS), and ultimately induces a state of “metabolic paralysis” in NK cells [79, 80]. Notably, this paralysis is not irreversible. Instead, intervening in distinct nodes of this circuit can achieve partial functional rescue [77, 78, 81]. For instance, while inhibiting FAO via carnitine palmitoyltransferase 1B (CPT1B) can restore glycolysis [77], paradoxically agonizing PPARγ [78] can increase the mitochondrial potential, underscoring that lipid accumulation itself is a maladaptive endpoint that leads cells to be in an inflexible state. Crucially, the inhibitory effects extend beyond provisional metabolic disturbances. In lipid-rich microenvironments, dysfunction persists even after stimulus removal, as signals, such as oleic acid suppress histone acetyltransferase expression via PPARδ, reducing H3K27 acetylation at effector gene loci and establishing long-term epigenetic silencing of NK cell functions [82].

Another critical vulnerability lies in the lipid metabolic processes that maintain plasma membrane functionality. Abnormalities in membrane topology and lipid raft integrity, which are caused by deficits in sphingomyelin synthesis [83] and cholesterol uptake [84, 85], directly impair the formation of cytolytic immunological synapses. Furthermore, the uptake of TME-derived polar lipids, such as phosphatidylcholine (36:1), in ovarian cancer ascites alters the expression of enzymes involved in the Lands cycle and further perturbs membrane order [86]. This disruption impairs the capacity of NK cells to polarize toward target cells and form targeted immune synapses, thereby crippling cytotoxicity. Together, different layers of metabolic reprogramming, including energy metabolism and membrane biophysics, converge to suppress the antitumor response of NK cells within the TME.

#### TAMs

Compared with their normal counterparts, TAMs have a higher lipid content, which is positively correlated with cancer progression. CD36-mediated lipid uptake promotes the storage of fatty acids, mitochondrial FAO and OXPHOS, supporting an M2-like, protumor phenotype characterized by the upregulation of anti-inflammatory effectors, such as CD206, arginase-1 (ARG1), IL-10 and TGFβ [87–91]. This metabolic program is fundamentally orchestrated by the transcription factor STAT6 in coordination with PPARγ [87, 89, 91]. In specific contexts, such as p53-deficient HCC, this pathway can be initiated by IL-34 signaling via the colony-stimulating factor-1 receptor (CSF1R) [90]. Additionally, intracellular fatty acid trafficking, facilitated by chaperones, such as FABPs, delivers activating ligands to PPARγ, which upregulates the expression of effectors, such as PD-L1, fine-tuning protumor functions [89, 92, 93]. In addition to scavenging exogenous lipids, lipogenesis promotes the metabolic fitness and persistence of TAMs [94, 95]. This is exemplified in PPARγ inhibitor-resistant breast cancer, where induced SREBP1 activity shifts metabolism from glycolysis to FAO, upregulating CSF1R in differentiating macrophages [94]. Alternatively, intracellular lipid pools and immunosuppressive phenotypes can originate from sphingolipid metabolism and membrane lipid reshuffling [96, 97]. For instance, ceramide accumulation driven by neutral ceramidase deficiency promotes TREM2^+^ macrophage differentiation, enhancing lipid droplet turnover and FAO to orchestrate CD8^+^ T-cell exhaustion. In HCC, phospholipase A2 activity contributes to subsequent DGAT-mediated droplet formation, which increases CCL20 secretion to recruit CCR6^+^ Tregs [97]. Furthermore, tumor-derived glucosylceramide drives phosphatidylcholine reshuffling, which induces PD-L1 and ARG1 upregulation through the ER stress response [96].

Similarly, cholesterol metabolism reshapes the functional fates of TAMs. Cholesterol efflux, mediated by ATP-binding cassette (ABC) transporters, such as ABCA1 and ABCG1, sustains alternative activation signaling in M2-like TAMs by reshaping lipid rafts [98]. In HCC, ABCA1 activity underpins the protumor phenotype of SPP1^+^ TAMs, whereas blocking cholesterol export reprograms them toward an immunostimulatory phenotype [99]. Conversely, cholesterol overload primarily impairs the antitumor functions of macrophages, as exemplified by glioblastoma-associated macrophages [100, 101]. The scavenging of myelin debris via CD36 drives the accumulation of cholesterol precursors, leading to metabolic and epigenetic reprogramming that silences proinflammatory and antigen-presentation pathways [100]. In monocyte-derived TAMs exposed to excessive amounts of oxysterols, failed compensatory cholesterol export triggers mitochondrial dysfunction and the upregulation of Siglec-10 and PD-1 expression, thereby suppressing phagocytosis capacity [101]. Additionally, oxidative stress dysregulates cholesterol homeostasis in microglia via SQLE upregulation, resulting in a protumor, anti-inflammatory phenotype with impaired antigen-presentation capacity [102]. CH25H-mediated oxidation and CYP11A1-driven side chain cleavage further entrench the immunosuppressive landscape. Intracellularly, 25-hydroxycholesterol potentiates STAT6 activity via the mTORC1/AMPK signaling pathway, promoting M2-like polarization [103]. Moreover, TAM-secreted glucocorticoids directly drive CD8^+^ T-cell exhaustion [104]. Thus, TAMs leverage the plasticity of lipid metabolism to promote their own survival and functional persistence while simultaneously generating bioactive lipids that disable local antitumor immunity.

#### Other immune cells

Lipid metabolism reprogramming modulates the functions of conventional CD4^+^ T cells. Like CD8^+^ T cells, conventional CD4^+^ T cells are vulnerable to lipid metabolism–related mitochondrial dysfunction, which undermines their survival, proliferation, and effector functions [105, 106]. For example, increased uptake and trafficking of linoleic acid induce lipid peroxidation and apoptosis in CD44^-^ CD4^+^ T cells [105]. Additionally, dysregulation of specific metabolic pathways can potentiate the protumor functions of CD4^+^ T cells. In melanoma, IL-4 signaling promotes steroidogenesis mainly in Th2 cells by upregulating CYP11A1 expression, thereby reinforcing an immunosuppressive microenvironment [107]. In nasopharyngeal carcinoma, the CD70–CD27 interaction activates a lipid metabolic network centered on lipid synthesis and mitochondrial FAO in stem-like CD4^+^ T cells, thus driving their differentiation into Tregs [108].

Lipid metabolism reprogramming serves as a cornerstone for FOXP3^+^ Treg functions and stability within the TME. For instance, the SREBP-dependent transcriptional program, potentiated by TCR stimulation in the TME, coordinates de novo lipogenesis and the mevalonate pathway to promote functional maturation and stability [109]. Concurrently, Tregs actively utilize CD36 [110] to acquire fatty acids and optimize mitochondrial fitness via PPARδ signaling. This sustains lactate metabolism and maintains the NAD^+^/NADH balance, thereby supporting survival [111]. Nevertheless, tumor-infiltrating Tregs can adapt to or even overcome metabolic perturbations [112, 113]. For example, acute mitochondrial dysfunction caused by FABP5 suppression upregulates IL-10 expression via the mtDNA-cGAS-STING pathway, enhancing the immunosuppressive potency of Tregs [112]. In lung cancer, Tregs outcompete CD8^+^ T cells in cholesterol uptake via the cholesterol acyltransferase SOAT2, thereby suppressing antitumor immunity, albeit at the cost of the proliferative capacity and FOXP3 stability of Tregs [113].

Like TAMs, other myeloid populations, such as MDSCs and tumor-associated neutrophils, also potentiate immunosuppressive activity by optimizing lipid metabolic programs. For example, in monocytic MDSCs, PPARα-driven FAO upregulates PD-L1 and ARG1 expression, contributing to acquired resistance to sorafenib in patients with HCC [114]. In neutrophils infiltrating liver metastases of CRC, enhanced DGAT1/2 activity promotes lipid droplet biogenesis, which mitigates oxidative stress and promotes neutrophil survival [115]. However, disruption of lipid homeostasis in these populations can inadvertently suppress antitumor immunity. Ferroptotic MDSCs and neutrophils have been shown to potently suppress CD8^+^ T-cell activity through the release of PGE2 and oxidized lipids [116, 117]. Specifically, in hypoxic polymorphonuclear MDSCs, upregulation of the fatty acid transporter FATP2 promotes the uptake of peroxidation-sensitive arachidonic acid (AA), whereas in neutrophils with decreased expression of the lysophospholipid acyltransferase MBOAT1, the accumulation of PUFA-containing phosphatidylethanolamine causes this metabolic vulnerability. The impact of lipid metabolism extends beyond myeloid cell–intrinsic changes to reshape the intercellular metabolic interplay within the TME. In CRC, for example, cholesterol competition between CD16^+^ neutrophils and NK cells, accompanied by the release of neutrophil extracellular traps, further promotes NK cell death [85].

In stark contrast to these protumor myeloid subsets, the immunostimulatory capacity of dendritic cells (DCs) is frequently undermined through TME-induced lipid dysregulation. For example, HCC-derived α-fetoprotein (AFP) skews DCs toward glycolysis dependency while suppressing fatty acid metabolism and impairing functional maturation and the expression of costimulatory ligands [118]. On the other hand, excessive uptake of extracellular fatty acids dampens the antigen-processing and antigen-presenting capacity of DCs, severely impairing T-cell activation in malignancies, such as ovarian cancer [119]. Oxysterols, such as 27-hydroxycholesterol, further compromise DC function by promoting cholesterol efflux, which downregulates the expression of surface markers, such as the chemokine receptor CCR7 [120, 121]. In a more transformative manipulation, lactate can reprogram DCs into a mature regulatory phenotype (CD63^+^ mregDCs) by bolstering FAO and cholesterol synthesis, endowing them with direct immunosuppressive activity [122].

### Lipid metabolism reprogramming in stromal cells

Lipid metabolism reprogramming is increasingly recognized as a key driver of the functional plasticity and heterogeneity of cancer-associated fibroblasts (CAFs) in the TME. One pathway involves the differentiation of precursor cells into pro-tumor CAF subsets. For instance, in HCC, tumor-derived glycoproteins synergize with IL-6 signaling to activate cholesterol synthesis in hepatic stellate cells (HSCs), driving their differentiation into α-SMA^+^ CAFs that foster cancer stem cell niches [123]. Alternatively, CAFs can evolve into a lipid-rich, adipocyte-like phenotype under the influence of tumor-driven proadipogenic factors, such as the bone morphogenetic protein BMP2/4, characterized by upregulated lipid droplet–associated proteins and markers for adipocytes, such as transcription factor 21 [124, 125]. The metabolic output of these lipid-loaded CAFs is central to their protumor functions. Specifically, they can directly transfer lipid vesicles to fuel tumor growth and secrete vascular endothelial growth factor A to accelerate angiogenesis [124, 125]. Furthermore, CAFs elicit immunoregulatory effects in the TME. A distinct subset of HSC-derived CAFs with enriched cholesterol metabolism upregulate macrophage migration inhibitory factor upon CD36-mediated uptake of oxidized LDL (oxLDL), facilitating the recruitment of MDSCs [126]. Thus, lipid metabolism reprogramming enables CAFs to execute specialized functions that drive tumor progression.

Other stromal cells, such as cancer-associated adipocytes [127–130] and endothelial cells [131], are also reprogrammed by TME factors. Specifically, inflammatory mediators, such as IL-6 [129] and tumor-derived metabolic signals, such as fibroblast growth factor 21 [127] and adrenomedullin [128] can activate lipolysis in adipocytes, leading to the substantial release of fatty acids. This metabolic shift is often coupled with dedifferentiation toward a fibroblast-like, proinflammatory state [128, 130]. These dedifferentiated adipocytes exhibit decreased expression of core adipogenic regulators but significantly upregulated expression of proinflammatory adipokines, such as IL-6 and IL-8, further reinforcing tumor-promoting metabolic dysregulation in the TME [128–130, 132]. These findings highlight that the lipid metabolic circuitry within stromal cells represents a central orchestrator of their identity and protumor capacity, directly fueling tumor cell proliferation, angiogenesis, and immunosuppression.

### Lipid metabolism regulates the communication between tumor cells, immune cells, and stromal cells

In the TME, lipid metabolism reprogramming orchestrates intercellular crosstalk. As central architects, tumor cells drive immune evasion by dictating local lipid availability [133–135], such as by starving CD8^+^ T cells through competitive uptake [133] or, conversely, by relinquishing extracellular lipids to immunosuppressive populations [134]. Simultaneously, they remodel intrinsic lipids to resist cytotoxicity, specifically by depleting PUFA-containing phospholipids to evade CD8^+^ T-cell–induced ferroptosis [38]. Furthermore, tumor-intrinsic lipid metabolism not only modulates immune interactions through inflammatory mediators and surface proteins but also releases metabolites that selectively attract immunosuppressive cells while compromising antitumor immunity. In turn, immune cells and stromal cells are not mere passive recipients but actively contribute to this metabolic dialog by providing lipid nutrients and signaling molecules, thereby supporting tumor progression.

#### Tumor cells as drivers of immunomodulation via lipid metabolism

**Fig. 2:**
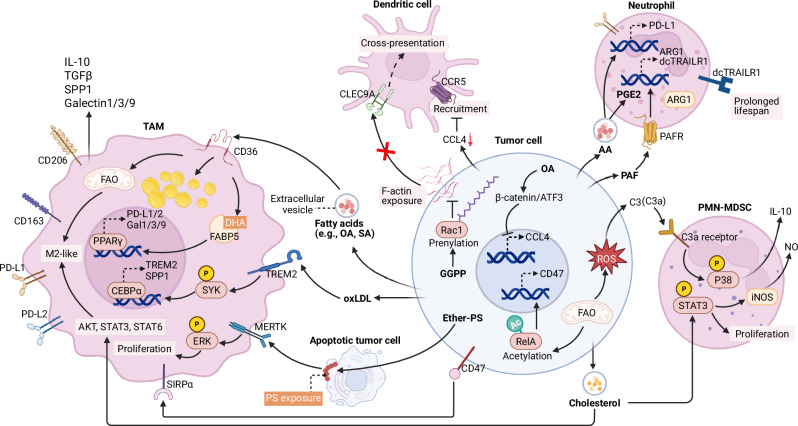
Lipid metabolism in tumors drives the immunosuppressive reprogramming of myeloid immune cells in the TME. At the membrane interface, lipid-driven signaling, such as FAO-dependent CD47 upregulation and ether-PS exposure, involves specific myeloid receptors (e.g., SIRPα and MERTK) to evade phagocytosis and support TAM proliferation. Within the extracellular milieu, tumor-derived inflammatory mediators (e.g., C3a) and bioactive lipids (e.g., PAF and oxLDL) activate downstream pathways to induce protumor phenotypes in TAMs, PMN-MDSCs and neutrophils. Furthermore, tumor-secreted fatty acids promote M2-like polarization of TAMs by fueling FAO and activating PPARγ signaling. Collectively, this metabolic crosstalk fundamentally reshapes the recruitment and functional identity of multiple myeloid lineages in the TME. Created at https://BioRender.com. Abbreviations: AA arachidonic acid, ARG1 arginase 1, ATF3 activating transcription factor 3, C3 complement 3, CCL4 C-C motif chemokine ligand 4, CLEC9A, C-type lectin domain containing 9A, dcTRAIL-R1 decoy TRAIL receptor 1, Ether-PS ether-phosphatidylserine, GGPP geranylgeranyl pyrophosphate, iNOS inducible nitric oxide synthase, MERTK MER proto-oncogene, tyrosine kinase, OA oleic acid, oxLDL oxidized low-density lipoprotein, PAF platelet-activating factor, PAFR platelet-activating factor receptor, PGE2 prostaglandin E2, PMN-MDSC polymorphonuclear myeloid-derived suppressor cell, Rac1 Ras-related C3 botulinum toxin substrate 1, SA stearic acid, SIRPα signal regulatory protein alpha, SPP1 osteopontin, TAM tumor-associated macrophage, TREM2 triggering receptor expressed on myeloid cells 2

**Fig. 3:**
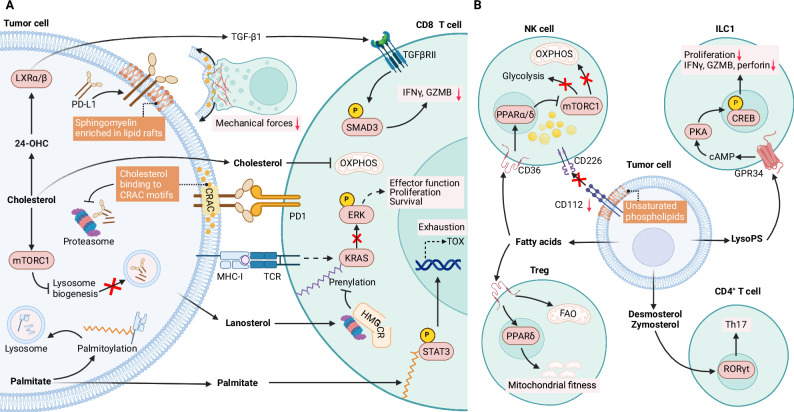
Tumor lipid metabolism modulates the functional fate of lymphoid immune cells in the TME. **A** Lipid-driven crosstalk between tumor cells and CD8^+^ T cells. Lipid metabolism in tumors impairs CD8^+^ T-cell–mediated immunity across multiple spatial and molecular dimensions. At the physical interface, altered lipid profiles promote the lysosomal degradation of MHC-I via palmitoylation, stabilize PD-L1, and attenuate mechanotransduction at the immunological synapse. Within the extracellular milieu, 24-hydroxycholesterol stimulates TGF-β1 secretion to further suppress T-cell activity. Intracellularly, exposure to a lipid-rich microenvironment induces CD8^+^ T-cell dysfunction by triggering mitochondrial dysfunction, impeding TCR signaling via lanosterol, and accelerating progressive exhaustion through aberrant STAT3 palmitoylation. **B** Tumor-derived lipids coordinate diverse outcomes across the broader lymphoid compartment. Specific lipid species disrupt energy metabolism and membrane recognition to evade natural killer (NK) cells, release lysophosphatidylserine (LysoPS) to dampen type 1 innate lymphoid cell (ILC1) activity, skew CD4^+^ T-cell differentiation toward Th17 cells, and serve as nutrient sources to sustain the protumor functions of regulatory T cells (Tregs). Created at https://BioRender.com. Abbreviations: 24-OHC 24-hydroxycholesterol, CRAC cholesterol-recognition amino acid consensus motif, CREB cAMP response element-binding protein, ERK extracellular signal-regulated kinase, FAO fatty acid oxidation, GPR34 G protein-coupled receptor 34, GZMB granzyme B, HMGC 3-hydroxy-3-methylglutaryl-CoA reductase, IFNγ interferon gamma, ILC1 type 1 innate lymphoid cell, KRAS Kirsten rat sarcoma viral oncogene homolog, LXR liver X receptor, LysoPS lysophosphatidylserine mTORC1 mechanistic target of rapamycin complex 1, NF-κB nuclear factor kappa-light-chain-enhancer of activated B cells, PD-1 programmed cell death protein 1, RORγt retinoic acid receptor-related orphan receptor gamma t, SMAD3 suppressor of mothers against decapentaplegic homolog 3, TGF-β1 transforming growth factor-beta 1, TGFβRII transforming growth factor-beta receptor II, TOX thymocyte selection-associated high mobility group box

Lipid metabolism reprogramming in tumor cells extensively shapes their interplay with both myeloid (Fig. 2) and lymphoid (Fig. 3) immune cells, fostering an immunosuppressive niche that facilitates evasion of immune destruction. To reshape the physical and functional interface exposed to immune cells, lipid metabolism primarily alters surface protein landscapes and membrane biophysical properties. For example, FASN-derived palmitate targets MHC-I for lysosomal degradation via palmitoylation, disrupting CD8^+^ T-cell recognition [136]. Melanoma SCD activity increases membrane fluidity while downregulating the expression of the activating ligands CD112 and CD155, leading to the evasion of NK cell–mediated death [137]. Glioblastoma utilizes FAO to promote NF-κB activation via acetylation, transcriptionally upregulating the CD47 “do not eat me” signal to resist phagocytosis [138]. Like altered fatty acid metabolism, changing levels of membrane phospholipids similarly drive immunomodulation. For example, in breast cancer cells, the expression of the phosphatidylserine synthase PTDSS1 is upregulated to produce ether-phosphatidylserine, which activates macrophage MERTK during apoptosis to promote TAM proliferation [139]. In pancreatic cancer, the upregulation of sphingomyelin synthase 2 drives sphingomyelin production, thereby reshaping the lipid composition of membrane microdomains and enriching the PD-L1 protein in lipid rafts [140]. Furthermore, cholesterol synthesis promotes PD-L1 protein stabilization through posttranslational mechanisms [141, 142], and cholesterol enrichment in the tumor cell plasma membrane attenuates mechanical force transduction at immunological synapses [143], together inducing CD8^+^ T-cell dysfunction. Additionally, GGPP-driven prenylation of the GTPase Rac1 prevents cytoskeletal exposure, thereby preventing detection by DCs [144].

Tumor cells also actively manipulate the extracellular milieu, either by secreting soluble inflammatory mediators or by tuning the availability of bioactive lipids. In CRC, SCD1-derived MUFAs abrogate CCL4 expression via β-catenin signaling, impairing DC recruitment and stalling the cancer-immunity cycle [145]. In HCC, enhanced FAO drives complement C3 production, which subsequently activates PMN-MDSCs via the p38 MAPK pathway [146]. Furthermore, altered endosomal trafficking of cholesterol promotes its accumulation and oxidation into 24-hydroxycholesterol, which induces TGFβ1 secretion to suppress CD8⁺ T-cell activity [147]. More directly, tumors shed bioactive lipids to engage specific extracellular receptors on immune cells [135, 148–151]. In glioblastoma, for example, AA produced from 2-arachidonoylglycerol by monoacylglycerol lipase (MAGL) [151] can be converted into PGE2 to drive immunosuppression via G protein-coupled receptors (GPRs), such as EP4. In HCC, SQLE upregulation enhances local oxidative stress, leading to oxLDL accumulation, which induces the formation of protumor TREM2^+^ SPP1^+^ TAMs [148]. Phospholipids represent another major class of lipid mediators [135, 149, 150]. By upregulating the expression of the lipase ABHD16A, tumor cells release lysophosphatidylserine, which acts on GPR34 to downregulate the expression of antitumor effectors in type 1 innate lymphoid cells [150]. A novel example of metabolic competition is found in CRC, where tumor cells deplete microenvironmental lysophosphatidic acid (LPA) resources via lysophosphatidic acid acyltransferase 4. This impairs M1-like polarization through ablation of LPA receptor-mediated NF-κB signaling [135].

Furthermore, tumor-derived lipids can reshape antitumor immunity by rewiring immunometabolism or by acting as intracellular signaling modulators. While tumor-synthesized fatty acids [20, 152, 153] disrupt energy metabolism in NK cells, immunosuppressive immune cells, such as Tregs [20, 111] and M2-like TAMs [88, 152] readily utilize these lipids to sustain their protumor functions. Furthermore, immune suppression is fine-tuned by specific lipid species [92, 154, 155]. For instance, FASN-derived palmitate accelerates CD8^+^ T-cell exhaustion by promoting STAT3 palmitoylation, while the fatty acid elongase ELOVL5–synthesized AA can be exploited by neutrophils to generate PGE2, converting them into potent CD8^+^ T-cell suppressors [155]. Similarly, tumor-derived sterols reshape the immune landscape in the TME. Driven by enhanced synthesis in tumor cells, extracellular vesicle-carried cholesterol enhances the immunosuppressive potency of TAMs [156] and MDSCs [157, 158]. However, exposure to a cholesterol-rich TME, as exemplified in SQLE-high HCC, causes mitochondrial dysfunction and accelerates exhaustion in CD8^+^ T cells [157], while cholesterol precursors, such as lanosterol [159] impede the mevalonate pathway in CD8⁺ T cells, hindering TCR signaling. Moreover, zymosterol and desmosterol promote Th17 polarization and upregulate the production of granulocyte‒macrophage colony-stimulating factor (GM-CSF), underlying immune suppression in CRC with the upregulation of SQLE and the postlanosterol enzyme CYP51A1 [160]. Collectively, tumor cells exploit multifaceted lipid-mediated mechanisms to impair antitumor immunity and foster a protumor milieu.

#### Immune cells and stromal cells as lipid suppliers and metabolic hubs

Beyond their canonical roles in the TME, immune cells and stromal cells are often coopted by malignant cells to serve as direct suppliers of lipid metabolites. In the immune compartment, this metabolic support is exemplified by the manipulation of cholesterol efflux. In glioblastoma, mesenchymal-like tumor cells prime TAMs to engulf myelin debris, leading to cholesterol overload and subsequent efflux, which is then exploited by tumor cells [100]. Similarly, in EGFR-mutant lung cancer, tumor-derived GM-CSF activates PPARγ signaling in alveolar macrophages, enhancing cholesterol efflux, which sustains oncogenic EGFR signaling [161]. Even more aggressively, pancreatic cancer cells can directly extract cholesterol from CD8^+^ T cells via the cholesterol transporter NPC1L1, simultaneously acquiring lipids and impairing CD8^+^ T-cell activity [74]. Stromal cells also engage in lipid nourishment. For instance, a subset of lipogenic CAFs secretes a lipid profile rich in MUFAs and unsaturated phospholipids, alleviating ER stress and fueling energy metabolism in tumor cells [162, 163]. In therapy-resistant prostate cancer, STEAP4^+^ CAFs increase phosphatidylcholine synthesis, which activates HSP90/HIF1α signaling in tumor cells, promoting cancer cell stemness [164].

These relatively direct interactions often extend beyond simple bilateral exchange, giving rise to intricate, multidirectional metabolic circuits that coordinate multiple cell types. In primary breast cancer lesions, mitochondrial dysfunction in adipocytes induces IL-6–mediated lipolysis and the release of free fatty acids, which are then utilized by tumor cells and Tregs [129], simultaneously fueling malignancy and suppressing antitumor immunity. Such multiparty cross-talk is further elaborated in metastatic niches. In liver-metastatic CRC, IL-33 secreted by activated HSCs induces lipid droplet biogenesis in neutrophils, and these lipids then reactivate dormant tumor cells via enhanced FAO and eicosanoid synthesis [115]. A particularly sophisticated self-amplifying circuit has been revealed in lung-metastatic breast cancer, where lung mesenchymal cell (MC)–derived PGE2 promotes lipid accumulation in neutrophils through HILPDA-dependent suppression of ATGL [165]. These lipid-laden neutrophils not only supply lipids to cancer cells via extracellular vesicles but also secrete IL-1β, which sustains lipid accumulation and PGE2 production in lung MCs. In turn, lung MCs shuttle lipids to both tumor cells and NK cells, simultaneously promoting metastasis and suppressing immune surveillance [166], ultimately forming a self-reinforcing metabolic loop that fuels tumor progression.

## Targeting lipid metabolism for cancer immunotherapy

Lipid metabolism reprogramming in the TME offers compelling vulnerabilities for cancer immunotherapy. A notable milestone is the clinical translation of inhibitors targeting PCSK9, a classic regulator of cholesterol metabolism. While these agents demonstrate strong synergy with anti-PD-1 therapy across multiple malignancies and have advanced into phase II clinical trials, their immunomodulatory effects are largely independent of lipid lowering [167]. Instead, PCSK9 restricts tumor immunity via nonmetabolic interactions, specifically by targeting MHC-I for lysosomal degradation [168]. Consequently, interventions that genuinely rely on metabolic rewiring to reverse immunosuppression represent a distinct frontier, although evaluation of these interventions in combination with cancer immunotherapy remains largely restricted to preclinical interventions. Nevertheless, pharmacological inhibition of these canonical pathways, even as monotherapies, can substantially remodel the TME, potentially sensitizing tumors to immunotherapy. Table 1 summarizes the in vivo immune mechanisms elicited by these lipid metabolism inhibitors.

**Table 1:** Representative pharmacological agents targeting lipid metabolism to reprogram antitumor immunity

Inhibition strategy		Target	Drug	Immunological Mechanism	Ref
De novo lipogenesis		ACLY	EVT0185	Upregulation of tumor-derived CXCL13 to promote tertiary lymphoid structure formation	[169]
		ACLY	Bempedoic acid (BemA)	Induction of lipid peroxidation and subsequent cGAS-STING activation in tumor cells to enhance CD8^+^ T-cell infiltration while upregulating PD-L1 expression	[25]
		ACC1	ND-646	Promotion of mitochondrial respiration in in vitro activated and expanded effector CD8^+^ T cells and to enhance memory formation and polyfunctionality upon adoptive transfer	[59]
		FASN	Cerulenin	Alleviation of tumor-derived fatty acids to reprogram TAMs toward an M1-like phenotype	[152]
		FASN	C75	Alleviation of tumor-derived fatty acids to suppress STAT3 palmitoylation and terminal exhaustion in CD8^+^ T cells	[154]
		FASN	Orlistat, TVB-2640	Suppression of MHC-I palmitoylation to stabilize its membrane expression in tumor cells	[136]
Fatty acid desaturation		SCD1	A939572	Suppression of β-catenin-ATF3 signaling to upregulate the chemokine CCL4 in tumor cells Alleviation of ER stress to upregulate CCL4 in CD8^+^ T cells	[145]
Lipid transport		FABP1	Orlistat	Suppression of fatty acid metabolism and PPARγ signaling to reprogram TAMs toward an immunostimulatory phenotype	[89]
		FABP5	SBFI-26	Suppression of lipid accumulation, FAO, and PPARγ signaling to alleviate the pro-tumor functions in TAMs	[171]
		CD36	PLT012 (an anti-CD36 antibody)	Broad blockade of CD36-mediated lipid metabolism in CD8^+^ T cells, TAMs, and Tregs to restore tumor-killing capacity and alleviate immunosuppression.	[170]
		CD36	SSO	Suppression of oxLDL uptake by CAFs to attenuate MIF production and subsequent MDSC recruitment	[126]
FAO		CPT1/2	Perhexiline	Promotion of metabolic shift toward glycolysis to restore effector functions in effector CD8^+^ T cells	[63]
		CPT1	Etomoxir	Suppression of mitochondrial FAO to alleviate the pro-tumor functions in TAMs Alleviation of the NF-κB subunit RelA acetylation to suppress CD47 transcription	[87, 138]
Triacylglycerol synthesis		DGAT1	T863	Suppression of lipid accumulation to induce cell death and alleviate pro-tumor functions in TAMs	[97]
		DGAT2	PF-06424439	Suppression of lipid accumulation to induce apoptosis and alleviate pro-tumor functions in TAMs	[97]
Monoacylglycerol hydrolysis		MAGL	JZL184	Suppression of AA release from 2-arachidonoylglycerol to decrease PGE2, which skews TAMs toward M1-like phenotype	[151]
Cholesterol uptake		NPC1L1	Ezetimibe	Inhibition of tumor-NPC1L1–induced cholesterol efflux to restore TCR signaling in CD8^+^ T cells Restoration of costimulatory signaling in CD8^+^ T cells	[74]
Cholesterol synthesis		HMGCR	Simvastatin	Prevention of cholesterol overload to delay terminal exhaustion, and to prevent senescence in CD8^+^ T cells Attenuation of lysosomal cholesterol and mTORC1 activity to induce lysosome biogenesis and promote PD-L1 degradation in tumor cells Alleviation of GGPP to induce NLRP3-dependent immunogenic cell death (pyroptosis) in tumor cells	[21, 69, 72, 144]
		SQLE	Terbinafine	Regulation of cholesterol homeostasis to restore immunostimulatory phenotype in microglia Alleviation of tumor-derived cholesterol to suppress MDSCs and reinvigorate CD8^+^ T cells Restriction of local oxidative stress and oxLDL to suppress TAMs and reinvigorate CD8^+^ T cells	[102, 148, 157]
		CYP51A1	Ketoconazole	Alleviation of tumor-derived distal cholesterol precursors to restrict immunosuppressive immune cells infiltration and reinvigorate CD8^+^ T cells	[160]

*AA* arachidonic acid, *ACC1* acetyl-CoA carboxylase 1, *ACLY* ATP citrate lyase, *ATF3* activating transcription factor 3, *CAFs* cancer-associated fibroblasts, *CCL4* C-C motif chemokine ligand 4, *CD8* cluster of differentiation 8, *CD36* cluster of differentiation 36, *CD47* cluster of differentiation 47, *CPT2* carnitine palmitoyltransferase 2, *CXCL13* C-X-C motif chemokine ligand 13, *CYP51* cytochrome P450 family 51, *DGAT1* diacylglycerol O-acyltransferase 1, *DGAT2* diacylglycerol O-acyltransferase 2, *ER* endoplasmic reticulum, *FABP1* fatty acid binding protein 1, *FABP5* fatty acid binding protein 5, *FAO* fatty acid oxidation, *FASN* fatty acid synthase, *GGPP* geranylgeranyl pyrophosphate, *HMGCR* 3-hydroxy-3-methylglutaryl-CoA reductase, *MAGL* monoacylglycerol lipase, *MDSC* myeloid-derived suppressor cell, *MHC-I* major histocompatibility complex class I, *MIF* macrophage migration inhibitory factor, *mTORC1* mechanistic target of rapamycin complex 1, *NF-κB* nuclear factor kappa-light chain

Fatty acid metabolic pathways have provided promising druggable targets for treatment. Inhibiting de novo lipogenesis via ACLY inhibitors, such as EVT0185 [169] and bempedoic acid [25] sensitizes HCC to anti-PD-L1 therapy. Similarly, FASN inhibition reverses CD8⁺ T-cell exhaustion, thereby restoring the efficacy of PD-1 blockade in patients with HCC [136, 154]. Beyond de novo lipogenesis, SCD1 is another key target for overcoming therapeutic resistance. Combined treatment with the SCD1 inhibitor A939572 and anti-PD-1 antibody produces synergistic antitumor responses, resulting in complete tumor regression in a subset of CRC murine models [145]. In turn, CPTs represent another potential target, with perhexiline and etomoxir providing initial evidence that FAO fuels immunosuppression [63, 87, 138]. Additionally, orchestrators of lipid uptake, such as the transport proteins CD36 [90, 126, 170] and FABP5 [93, 171], have emerged as critical immunometabolic checkpoints. Remarkably, the humanized CD36 antibody PLT012 [170] exhibits broad immunometabolic modulation in HCC, significantly influencing the functional states of multiple immune cell types, including TAMs and CD8⁺ T cells.

In addition to fatty acid metabolism, diverse lipid pathways have emerged as compelling therapeutic targets. Dual inhibition of DGAT1 and DGAT2 reduces the number of lipid-laden TAMs in HCC, effectively limiting Treg recruitment and delaying in vivo tumor progression [97]. By blocking 2-arachidonylglycerol hydrolysis, the MAGL inhibitor JZL184 reduces PGE2 levels in the glioblastoma microenvironment, thereby suppressing cancer stem cell self-renewal and restoring antitumor immunity [151]. Cholesterol metabolism, in particular, offers a rich array of druggable targets. Repurposed FDA-approved agents, such as the HMGCR inhibitor statins [21] and the SQLE inhibitor terbinafine [102, 148, 157], reshape the TME into an immune-permissive state, resulting in notable synergistic efficacy with PD-1 blockade across distinct tumor types. Targeting postsqualene steps with the CYP51 inhibitor ketoconazole reduces distal CHOL precursors to reverse immunosuppression and impede tumor growth in mouse models [160]. Furthermore, blocking cholesterol transport via the NPC1L1 inhibitor ezetimibe reinvigorates pancreatic cancer immunity. This blockade liberates the costimulatory receptor ITGAL, driving robust T-cell activation and cytotoxicity.

## Conclusions and perspectives

Lipid metabolism reprogramming is a central driver of the immune landscape within the TME. Beyond directly conferring proliferative and survival advantages to tumor cells, this metabolic rewiring orchestrates widespread immune evasion. Specifically, aberrant lipid metabolism induces severe metabolic dysfunction and exhaustion in antitumor effector cells, notably CD8^+^ T cells and NK cells, while simultaneously activating and fueling immunosuppressive populations, such as TAMs. Beyond these cell-intrinsic alterations, lipid-mediated intercellular crosstalk synergistically perpetuates this hostile niche. Thus, dysregulated lipid metabolism fundamentally underpins tumor escape from immune surveillance and represents a key mechanism of immunotherapy resistance.

Current pharmacological agents targeting lipid metabolismreveal that blocking tumor–immune crosstalk and disrupting TAM lipid metabolism represent primary strategies to remodel the immunosuppressive microenvironment. Despite this potential, the clinical application of systemic metabolic interventions is often hindered by the profound heterogeneity of the TME and detrimental off-target effects. This is exemplified by the context-dependent effect of statins, which may inadvertently exacerbate cholesterol starvation in CD8^+^ T cells, leading to profound suppression of their activation and survival [73, 113]. Furthermore, statins can induce unexpected metabolic rewiring, such as by driving SREBP1/TGFβ signaling in KRAS-mutant pancreatic cancer models, thereby significantly enhancing tumor aggressiveness [172]. To overcome these bottlenecks, cell-specific and precise immunometabolic rewiring has emerged as a promising therapeutic strategy. For example, CD40 agonism reprograms TAMs via FAO- and ACLY-mediated epigenetic rewiring, enabling robust M1-like polarization even in a glucose-deprived TME [173]. Similarly, genetically engineering FOXP3 into CAR-T cells optimizes their intrinsic lipid utilization, conferring superior persistence and therapeutic efficacy [174].

The omics era has fundamentally transformed lipid metabolism research from descriptive observation to mechanistic discovery through the integration of multilayered technologies. Multiomics strategies now couple transcriptomics with metabolomics or lipidomics to directly correlate gene expression with metabolic flux. This integrated approach links transcriptional changes to functional metabolite loss [75] and pinpoints cholesterol accumulation in specific myeloid populations [158] as a key immunosuppressive mechanism. Proteomics extends this functional mapping by systematically profiling differential protein expression and identifying key metabolic regulators in both immune [99] and tumor cells [74]. Furthermore, by characterizing the protein cargo of extracellular vesicles [129], proteomics reveals the mechanisms underlying tissue-specific intercellular communication and distal metabolic reprogramming. More recently, single-cell and spatial technologies have resolved the heterogeneity and physical niches of lipid metabolism. Studies combining scRNA-seq with spatial transcriptomics have shown that lipid-laden macrophages colocalize with tumor cells in glioblastoma [100]. At an even finer resolution, single-cell mass spectrometry has directly connected membrane lipid composition to immune cell structural defects [83]. Collectively, these advances establish lipid metabolism as a dynamic, spatially organized intercellular communication network within the TME.

Investigations have now extended to subcellular resolution and dynamic intercellular lipid flux. Subcellular lipidomics has revealed organelle-specific lipid signaling, demonstrating that the composition of the lysosome [103] or ER membrane [96] directly regulates immune cell functions. Simultaneously, isotopic tracing [161] and extracellular vesicle analysis [155] have revealed direct lipid transfer between cells, establishing that metabolites serve as intercellular messengers rather than mere energy sources. Looking forward, integrating multimodal datasets represents the next critical frontier. The emergence of multimodal cohort studies that combine transcriptomics, imaging mass cytometry, and epigenetic data points to the development of personalized medicine in which metabolic signatures inform therapeutic strategies [140]. Thus, the omics era has established lipid metabolism as a key regulator of cell state transitions and intercellular communication. Harnessing this mechanistic understanding through continued technological innovation will be essential for developing novel cancer immunotherapies.
